# What Radiologist Should Know about MRI Translational Forces and Hazard: An Ex-Vivo Simulation of Retained Metallic Shrapnel

**DOI:** 10.1155/2021/6672617

**Published:** 2021-02-25

**Authors:** Ali Kanj, Ibrahim Ghosn, Assaad Mohanna, Georges Rouhana

**Affiliations:** ^1^Radiology Department, Hammoud Hospital University Medical Center, Saida, Lebanon; ^2^Fellow of Radiology, Radiology Department, Faculty of Medical Sciences, Lebanese University, Hadath, Lebanon; ^3^Radiology Department, Faculty of Medical Sciences, Lebanese University, Hadath, Lebanon

## Abstract

**Background:**

In a country immersed in endless rounds of wars, retained metallic foreign bodies remain a significant dilemma in the daily practice of every Lebanese radiologist. When a shrapnel's hazard is of concern, the decision between performing or refusing a justified MRI exam is not always straightforward. In this small trial, we aimed to better understand the shrapnel's MRI safety by mimicking our daily practice.

**Methods:**

Five shrapnel with an incremental increase in their long axis were put in an animal flesh and then introduced into a 3 T magnetic field. The behavior of each shrapnel was concretely assessed by performing before and after magnetic field exposure CT acquisitions.

**Results:**

Translation along the *z*-axis ranged from 0.9 mm to 2.8 mm. Torque angle ranged between 2.8 and 54 degrees with an average of 15.62 degrees.

**Conclusions:**

Shrapnel's movements in the magnetic field are not negligible during the acute phase of injury where there is no reinforcing fibroblastic reaction and invite us to reconsider the MRI safety of these metallic foreign bodies. Standard radiographs may be sufficient, but a targeted CT scan may be of better value for a confident decision for assessment of shrapnel position near viscera and major vessels.

## 1. Background

Lebanon, a more than 6 million population country [1], witnessed multiple consecutive wars and conflicts, generating hundreds of thousands of deaths and a higher number of injuries. After the recovery of material losses, war-related injuries and retained metallic foreign bodies are still buried in a considerable number of Lebanese people's organs. It could be secondary to explosive weapon-related fragments, Cluster Munitions, and the Anti-Personnel mine called shrapnel or gunshot-bonded bullets and pellets [2].

Surgical removal of the retained fragment from the human body is not always feasible or indicated. Surgeons usually decide by weighing benefits versus operation risk [3]. Therefore, the fate of these patients was to carry within their bodies these metallic fragments without problems until an MRI is judged necessary.

Almost on a daily basis, the Lebanese radiologists find themselves in a hard dilemma between the risk of hazardous incidence and the justified indication of MRI. Solving this dilemma is essential due to its medico-legal aspect and more importantly to insure patients' safety and optimal care [4]. With the advancement in MRI technology and the introduction of high field MRI systems as well as the increase of MRI implication in patient's management, the MRI safety issue should be re-evaluated or at least should be better understood.

To understand the factors influencing the MRI induced hazards, we present in this article a small ex-vivo trial of 5 different metallic shrapnel retained within an animal flesh simulating human extremity to assess the eventual torque and displacement in a 3 T MRI magnet.

## 2. Methods

Multiple studies are available in the literature concerning foreign bodies and MRI especially those performed to assess the MRI safety of metallic implants and orthopedic materials [5]. These studies were conducted under strict measures detailed by the American Society for Testing and Materials (ASTM). In this article we tried to simulate these methods with a simplified approach mimicking our daily practice.

### 2.1. Model

Knowing that the relative risk of injury is determined by the characteristics of the foreign body in question (ferromagnetic properties, geometry and dimensions) as well as by the strength of the static magnetic field, we tried to simulate a daily scenario when an MRI is ordered for a patient with a history of blast injury.

**Figure 1:**
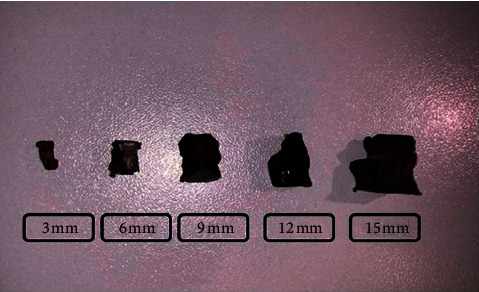
Shrapnel (dimensions of each long axis are shown).

- Steel shrapnel was cut into 5 smaller pieces of almost the same thickness but with a 3 mm incremental increase in the long axis (Figure 1).
- The size, volume, and weight of obtained shrapnel are shown in Table 1.
- An animal flesh was used as the container to simulate the consistency and the histology of the muscular compartment of a human extremity.

One shrapnel was introduced at a time during each trial.

### 2.2. Simulation

3 steps were performed consecutively:

- A baseline CT acquisition was performed using a GE 64-MDCT scan to assess the initial position and orientation of the fragment within the flesh.
- The model was put on the table of a 3T GE Discovery 750 W MRI and then slid through the magnet portal and toward the isocenter of MRI gantry. The model was then left for 5 min in the isocenter of the magnetic field and then slid back to the initial position through the magnet portal.
- A second CT acquisition was performed using the same CT machine and centering parameters to assess the new position and orientation of the shrapnel.

### 2.3. Measurements

All measurements were done on Advantage Window Workstation AW4.6 from GE.

- Pre- and postmagnetic field exposure CT images were fused, and a qualitative visual assessment of their rotation around their centers was done
- Shrapnel translation was measured relatively to the meat's plate
- Rotational angle (RA) was calculated on the fused images between the initial and new position of the long axis of each shrapnel along the *z*-axis of the magnet and the *xy* plane orthogonal to the *z*-axis

## 3. Results

The translation and the rotation of studied shrapnel are shown in Table 2.

### 3.1. Translation

The five shrapnel of incremental size difference experienced a subtle translation along the *z*-axis. Their translation ranged from 0.9 mm to 2.8 mm. The minimal translation was noted with the smallest shrapnel size (shrapnel *A*), whereas the maximum translation was seen with the shrapnel *D*. Only 0.5 mm difference is noted when comparing the translation of the shrapnel *D* to the largest shrapnel (shrapnel *E*).

### 3.2. Torque

All the five shrapnel experienced rotation along the *B*_0_ axis and in the *xy* plane.

**Figure 2:**
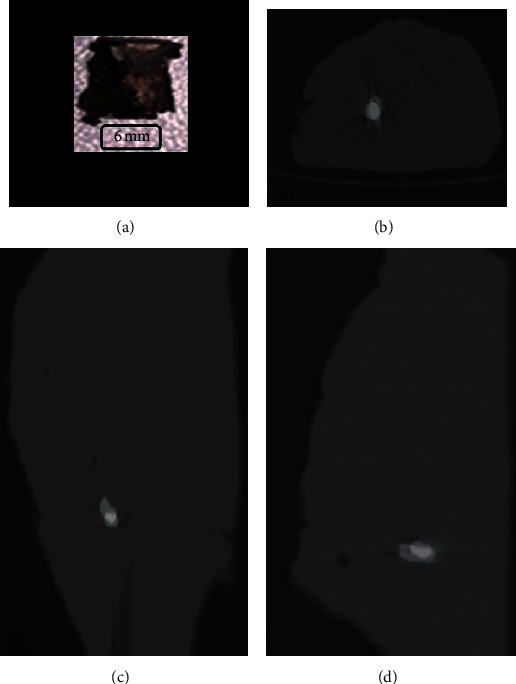
Shrapnel *B* (a). Fused images before and after MRI exposure in axial (b), coronal (c), and sagittal (d) plans.

We calculated the alignment within the *z*-axis by measuring, on the sagittal plane, the angle between the greater axis of the shrapnel and the greatest axis of the flesh (parallel to the *z*-axis). It ranged between 2.8 and 54 degrees with an average of 15.62 degrees (Figure 2).

The average rotation angle for the 5 shrapnel in the coronal plane was 72.18 degrees.

The average rotation angle of the five shrapnel in the *xy* plane was 54.26 degrees.

## 4. Discussion

Shrapnel is a major foreign body issue encountered in our daily practice especially in a country immersed in endless rounds of wars. It got its name from General Henry Shrapnel [6] of the British Army's Royal Artillery who invented during the Peninsular War an exploding shell that broke apart and shattered when it was detonated.

Because of their ferromagnetic potential, they are considered to be a relative contraindication to MRI exams [7]. However the word “relative” is somewhat confusing and absurd and carries a lot of interpretations.

We tried to better understand their behavior by simplifying the physics of interactions between the retained objects and the magnetic field and by mimicking our daily practice with this trial using readily available materials.

### 4.1. Magnetic Field Interaction with Retained Metallic Shrapnel

When a metallic object is placed in a magnetic field, it will be subject to a magnetic force. This force is due to different factors related to the magnetic field itself (strength and spatial gradient strength) and other factors related to the metallic object put in the field (volume, surface shape, and composition determining its ferromagnetic characteristics) [8].

The magnetic field strength used in our daily practice varies between 0.3 T and 3 T and corresponds to *B*_0_. The spatial gradient of the magnetic field *B*_0_ is a major parameter in determining the interaction force exerted over a ferromagnetic object. It is defined as the change in the strength of the magnetic field proportional to distance and is measured in Tesla per meter (T/m) or in Gauss per centimeter (G/cm), where (1 T/m = 100 G/cm). This magnetic force can be further divided into two kinetic forces: the translational force and the torque force. The former is responsible for the displacement of an object within a magnetic field (translation and diversion), and the latter is responsible for the object's rotation movement [9].

#### 4.1.1. Translational Force or Displacement (*F*)

The translational force (*F*) exerted on a ferromagnetic shrapnel of a certain volume *V* with magnetic susceptibility *X* is proportional to the product of the static field (*B*) and the spatial gradient [8]:

(1) $$\begin{array}{c} F\infty X\cdot VB\cdot \frac{\text{d}B}{\text{d}z}. \end{array}$$

According to this equation; the maximal amplitude of (*F*) is as close to the opening of the magnet bore.

In MRI magnet, the direction of the magnetic field vector (*B*_0_) is horizontal; in 3 T MRI systems, the highest gradient occurs at a position range from 10 to 30 cm from laser point locator.

In contrast to torque force (*T*), translational force or displacement is minimal at the isocenter of the magnetic field.

When evaluating an implant deflection (translation), this can be easy extrapolated to any ferromagnetic foreign body. The ASTM considers a deflection of less than 45° insignificant, because the magnetically induced deflection force is less than the force on the implant due to gravity (the weight of the implant). Therefore, we can consider that any risk induced by a similar ferromagnetic force is no greater than any risk imposed by normal daily activity in the Earth's gravitational field.

In this trial, all shrapnel presented a minimal degree of translation ranging from 0.9 to 2.8 mm.

#### 4.1.2. Rotational Force or Torque (*T*)

The rotational force on a shrapnel dipole (*μ*m) in a magnetic field *B* is equal to the vector product of *μ*m and *B*:

(2) $$\begin{array}{c} T=\mu \text{m}\cdot B. \end{array}$$

This explains why the torque force (*T*) is maximum where the strength of *B*_0_ itself is maximum [9] independent of the spatial gradient amplitude. Therefore, the maximum torque occurs within the magnet bore in the isocenter, and it is proportional to the field strength, *B*_0_, which is constant (3 T) in our study. Its amplitude decreases as the shrapnel moves out of the isocenter.

If shrapnel has longitudinal shape, it will be exposed to the twisting magnetic force or torque (*T*). This force tends to align the shrapnel to the field and this force magnitude will be related to the angle between the shrapnel's long axis and the field direction.

For this reason and for irregular shaped ferromagnetic shrapnel retained in the body, the torque force (*T*) may be the essential safety issue as compared to translational force (*F*) especially when present in the proximity of a major vessel or nerve.

### 4.2. MRI Safety and Shrapnel

Shrapnel lodged in human bodies are considered relative contraindication for MRI and therefore leaving the final decision for radiologists. In front of a similar scenario, we are obliged to perform a full survey starting with shrapnel localization, dating the exposure, weighing the risk versus the benefit of the MRI, and also considering the availability of other alternative exams.

At least two significant MRI hazardous morbidity and mortality were reported in the literature; one with metallic clip with secondary rupture and bleeding after exposure to the magnetic field [10] and the second one for ocular injury related to missed intraorbital foreign body [11].

Other than shrapnel in/near the ocular globe that constitute the clearest contraindication, the shrapnel position near viscera and major vessels should be a concern while accepting to perform an MRI exam. Standard radiographies may be sufficient, but a targeted CT scan may be of better value for a confident decision.

In this small ex-vivo trial, we aimed to realize a simulation of circumstances that we encounter in our daily practice by using animal flesh composed of muscles, fat, and fascia's and thereby histologically similar to human extremity.

Our primary limitation was the lack of surrounding scar tissue and fibrosis, a factor that could fix and retain the metallic shrapnel reducing the degree of translation and torque. And this factor could explain the paucity of reported hazards in our practice with patients reporting shrapnel exposure.

The second limitation is the small number of studied shrapnel. The aim of this study is to understand the physics of MRI/foreign bodies' interactions rather than extracting formulas and statistical analysis and to encourage a better investment in this contradictory yet important issue.

## 5. Conclusions

Shrapnel-related MRI safety will remain a daily issue in our practice; better understanding of its behavior in MRI is mandatory for optimal patients' safety. This article proved that shrapnel's movements in the magnetic field is not negligible at least during acute phase of injury where there is no reinforcing fibrotic matrix and invites us to reconsider the MRI safety of these metallic foreign bodies and to look to this issue with mistrust in our future decision making. This article also shed some light on the usefulness of CT scan as a readily available tool to assess the position of the shrapnel and its relation to the surrounding noble structures helping the radiologist to take a more straightforward decision.

## Figures and Tables

**Table 1:** Shrapnel characteristics.

	*A*	*B*	*C*	*D*	*E*
Shrapnel size (mm)	1 × 3	5 × 6	8 × 9	8 × 12	11 × 15
Shrapnel weight (g)	0.29	1.11	1.99	3.06	6.9
Shrapnel volume (mm^3^)	80	200	450	500	900

**Table 2:** Shrapnel translation and rotation.

Shrapnel	*A*	*B*	*C*	*D*	*E*
Translation (mm)	0.9	1.4	2.2	2.8	2.3

Rotation angle in coronal plane (degree)	88.3	49.2	73.2	85.4	64.8

Rotation angle in axial plane (degree)	90	88.7	10.9	53.6	28.1

Alignment along *B*_0_ (after exposure)	5	54	15.4	2.8	0.9

## Data Availability

The data used to support this study are available on request from the corresponding auhtor.

## Abbreviations

MRI: Magnetic resonance imaging
CT: Computed tomography
*T*: Torque force
*F*: Translation force
ASTM: American Society for Testing and Materials.

## References

[1] The World Bank (2020). *Population, Total—Lebanon*.

[2] Geneva International Centre for Humanitarian Demining (2017). Explosive weapon effects. https://www.gichd.org/en/resources/publications/detail/publication/explosive-weapon-effects/.

[3] Guermazi A., Hayashi D., Smith S. E., Palmer W., Katz J. N. (2013). Imaging of blast injuries to the lower extremities sustained in the Boston marathon bombing. *Arthritis Care & Research*.

[4] MRI Safety (2020). Pellets, Bullets, and Shrapnel. http://www.mrisafety.com/SafetyInformation_view.php?editid1=192.

[5] McComb C., Allan D., Condon B. (2009). Evaluation of the translational and rotational forces acting on a highly ferromagnetic orthopedic spinal implant in magnetic resonance imaging. *Journal of Magnetic Resonance Imaging*.

[6] Shrapnel H. (2020). *Encyclopædia Britannica*.

[7] UCSF Radiology (2020). Relative contraindications. https://radiology.ucsf.edu/patient-care/patient-safety/mri/relative-contraindications#accordion-shrapnel.

[8] Panych L. P., Madore B. (2018). The physics of MRI safety. *Journal of Magnetic Resonance Imaging*.

[9] Shokrollahi P., Drake J., Goldenberg A. (2017). Quantification of force and torque applied by a high-field magnetic resonance imaging system on an ultrasonic motor for MRI-guided robot-assisted interventions. *Actuators*.

[10] McRobbie D., Moore E., Graves M., Prince M. (2017). *MRI from Picture To Proton*.

[11] Mamas N., Andreanos K., Brouzas D. (2018). Acute ocular pain during magnetic resonance imaging due to retained intraocular metallic foreign body: the role of ultrasonography and ultrasound biomicroscopy in diagnosis and management of this condition. *Journal of Ultrasound*.

